# Drug safety profiles in geriatric patients with Parkinson’s disease using the FORTA (Fit fOR The Aged) classification: results from a mono-centric retrospective analysis

**DOI:** 10.1007/s00702-020-02276-x

**Published:** 2020-12-01

**Authors:** S. Greten, J. I. Müller-Funogea, F. Wegner, G. U. Höglinger, N. Simon, U. Junius-Walker, S. Gerbel, O. Krause, M. Klietz

**Affiliations:** 1 Department of Neurology, Hannover Medical School, Carl-Neuberg-Straße 1, 30625 Hannover, Germany; 2 Department of General Practice, Hannover Medical School, Carl-Neuberg-Straße 1, 30625 Hannover, Germany; 3 Centre for Information Management (ZIMT), Hannover Medical School, Carl-Neuberg-Straße 1, 30625 Hannover, Germany

**Keywords:** Parkinson’s disease, Geriatric patients, Polypharmacy, Multimorbidity, FORTA prescribing list, Drug safety

## Abstract

To reduce potentially inappropriate medications, the FORTA (Fit fOR The Aged) concept classifies drugs in terms of their suitability for geriatric patients with different labels, namely A (indispensable), B (beneficial), C (questionable), and D (avoid). The aims of our study were to assess the medication appropriateness in PD inpatients applying the FORTA list and drug-drug interaction software, further to assess the adequacy of FORTA list for patients with PD. We retrospectively collected demographic data, comorbidities, laboratory values, and the medication from the discharge letters of 123 geriatric inpatients with PD at the university hospital of Hannover Medical School. Patients suffered on average from 8.2 comorbidities. The majority of the medication was labeled A (60.6% of PD-specific and 40.9% of other medication) or B (22.3% of PD-specific and 26.9% of other medication). Administered drugs labeled with D were amantadine, clozapine, oxazepam, lorazepam, amitriptyline, and clonidine. Overall, 545 interactions were identified, thereof 11.9% severe interactions, and 1.7% contraindicated combinations. 81.3% of patients had at least one moderate or severe interaction. The FORTA list gives rational recommendations for PD-specific and other medication, especially for general practitioners. Considering the demographic characteristics and the common multimorbidity of geriatric PD patients, this study underlines the importance of awareness, education, and preventive interventions to increase drug safety.

## Introduction

Parkinson’s disease (PD) is the second most common neurodegenerative disease. PD patients suffer from various motor and non-motor symptoms with profound impairments in quality of life (Valkovic et al. 2014; Kadastik-Eerme et al. 2015; Skorvanek et al. 2015; Klietz et al. 2018, 2020). Most of these patients are elderly and accordingly have several comorbidities (Klietz et al. 2019). Common comorbidities in PD are cardiovascular and cerebrovascular diseases, diabetes, polyneuropathy, frailty, sarcopenia, and atrial fibrillation (Klietz et al. 2018, 2019). The pharmacological treatment of PD in multimorbid patients usually results in polypharmacy (Klietz et al. 2019). Inappropriate interactions due to polypharmacy may cause drug-related hospital admissions (Budnitz et al. 2011; El Morabet et al. 2018; Thevelin et al. 2019), increase morbidity and mortality (Thevelin et al. 2019), and lead to higher health-care costs (Ernst and Grizzle 2001; Leendertse et al. 2011).

Tools to increase drug safety in geriatric patients with PD are urgently needed. There are some pre-existing classifications to detect potentially inappropriate medications (PIM) in geriatric patients (Beers et al. 1991; Gallagher et al. 2008; Holt et al. 2010). The FORTA (Fit fOR The Aged) concept categorizes drugs based on expert opinion into four different labels ranging from A (indispensable), B (beneficial), C (questionable) to D (avoid) (The FORTA authors/expert panel members et al. 2014). The FORTA list was internationally validated in a Delphi consensus method, confirmed in several prospective studies (Wehling et al. 2016; Pazan et al. 2018, 2019, 2020), and is updated regularly. Unfortunately, neither the FORTA list nor any other PIM tools are specifically designed for patients with PD (Beers 1991; Gallagher et al. 2008; Holt et al. 2010; The FORTA authors/expert panel members et al. 2014).

The aim of the present study was to analyze comorbidities, prescription patterns and drug safety in geriatric PD patients admitted to a tertiary hospital. We utilized the FORTA list to examine the appropriateness of the medication as well as drug-drug interaction software. Moreover, we assessed the FORTA list according to its adequacy and coverage of recommendations for PD drugs.

## Patients and methods

### Ethics

This retrospective mono-centric study, which was carried out in a tertiary hospital, was approved by the local ethics committee (No. 8939_BO_K_2020). All patients had given their written informed consent for retrospective analysis of their treatment in terms of anonymous hospital data.

### Data acquisition

Patients aged 70 years or older, with multimorbidity and polypharmacy were defined as “geriatric” according to the German Geriatric Society (Sieber 2007). Multimorbidity was defined as having three or more active diseases requiring medical treatment. Polypharmacy was defined as the use of five or more different long-term drugs. Patients were automatically identified for the study by the Enterprise Clinical Research Data Warehouse of the Hannover Medical School comprising clinical data of > 2.2 million patients (Gerbel et al. 2019). PD patients from the neurological department of Hannover Medical School with a hospital admission between 1st of January 2015 and 31th of December 2018 were included in our study. Medical data were gathered from the medical discharge letters and from the clinical meta-database of the hospital. Patients with atypical Parkinsonism, undefined Parkinsonism, secondary Parkinsonism, and vascular Parkinsonism as well as patients without sufficient informed consent were excluded from the study.

### Analysis

Demographic data (age, gender, duration of disease, Hoehn und Yahr scale (Hoehn and Yahr 1967)), comorbidities, pathological laboratory values, and medication were collected from the medical discharge letters. The duration of disease was defined as the time from the onset of first motor symptoms or, if not available, the time from the first diagnosis. PD-related accompanying symptoms or diseases (incontinence, psychosis, depression, orthostatic dysfunction, and dementia) were evaluated separately. The pathological laboratory values were analyzed and added to the comorbidities (e.g. long-term hypo-/hypernatremia or chronic renal failure), which enabled more accurate identification of contraindications. The comorbidities were classified according to the first level of ICD-10. Moreover, the most common comorbidities on the second level of ICD-10 were collected. Acute, transient diseases (e.g. infections or acute electrolyte imbalances) and their drug therapy were not included because of their mainly transient nature. Lastly, the Charlson Comorbidity Index (CCI) was calculated as a prognostic index for the 10-year survival of a patient (Charlson et al. 1987).

The discharge medication was divided into PD-specific drugs, non-oral PD-specific treatments, and other drugs. Using the FORTA list, every drug was assigned to a label according to its main treatment indication. These labels ranged from A (indispensable), B (beneficial), C (questionable) to D (avoid) (The FORTA authors/expert panel members et al. 2014). If the main indication of a drug was not clear or if there were competing indications (e.g. hypertension and heart failure), the drug would be assigned to the most favorable label for the individual drug. For example, beta-blockers are labeled B for the treatment of hypertension and A for the treatment of heart failure. The drug would be assigned to label A, if the patient suffered from both diseases or if the indication was not clear in the letter of discharge. If the main indication of a drug was not considered in the FORTA list, the drug would be classified as “no label”. This procedure was also applied to drugs with no disease-specific recommendation in the FORTA list (e.g. prednisolone for the treatment of autoimmune diseases). Contraindications were determined using the drug-specific specialist information. Potential drug-drug interactions (DDIs) were identified with the tool “Medibox” of the clinical decision support system AiD*Klinik*^®^ (AID, version 01.05.2020; Dosing GmbH, Heidelberg, Germany). The analysis did not include whether these DDIs resulted in individual side effects. DDIs were classified according to their severity ranging from “disputed evidence”, “evidence for no interaction”, “light interaction”, “moderate interaction”, “severe interaction” or “contraindicated combination”.

### Statistical analysis

Descriptive statistical analyses were performed with GraphPad Prism 5 (GraphPad Prism Software Inc., San Diego, California, USA) and Microsoft Excel 2010 (Microsoft Corporation, Redmond, Washington, USA). If applicable, data were reported as mean and standard deviation.

## Results

### Patient characteristics

123 geriatric patients with PD were included in the study. The basic demographic and clinical characteristics of the study participants are shown in Table 1. 78 patients were admitted to hospital for PD-related problems (e.g. adjustment of medication, worsening of symptoms), 43 for other neurological diseases (e.g. stroke, seizure), and two for other diseases (myocardial infarction, basal-cell carcinoma surgery). All patients were treated either from the beginning or during the course in a neurological ward. The mean age was 78.1 ± 4.9 years and the mean PD duration 10.3 ± 7.5 years. All Hoehn and Yahr stages were represented in the study sample. 61% of the patients were in an advanced disease stage (Hoehn and Yahr stage ≥ III). For some patients, the disease duration (*n* = 45; 36.6%) and Hoehn and Yahr stage (*n* = 40; 32.5%) were not available in their discharge letters. The average score of the Charlson Comorbidity Index was 4.9 ± 1.7, which corresponds to a mean estimated 10 year-survival of 33.1 ± 27.3%. A significant proportion of the patients suffered from PD-related accompanying diagnoses, like temporary psychosis (27.6%), dementia (25.2%), urinary incontinence (18.7%), depression (17.9%), and orthostatic hypotension (14.6%).

**Table 1:** Clinical and demographic characteristics of the PD patients (*n* = 123)

Characteristic	Value	
	Mean	SD
Age (years)	78.1	4.9
Disease duration (years)	10.3	7.5
Charlson Comorbidity Index		
Points	4.9	1.7
Estimated 10-year survival (%)	33.1	27.3

Characteristic	Value	
	*n*	%
Gender		
Female	48	39.0
Male	75	61.0
Hoehn & Yahr Stage		
I	1	0.8
II	7	5.7
III	32	26.0
IV	29	23.6
V	14	11.4
Not available	40	32.5
Dementia		
Yes	31	25.2
No	92	74.8
Psychosis (temporary)		
Yes	34	27.6
No	89	72.4
Depression		
Yes	22	17.9
No	101	82.1
Orthostatic dysfunction		
Yes	18	14.6
No	105	85.4
Incontinence		
Yes	23	17.8
No	100	81.3

### Frequent comorbidities

**Fig. 1:**
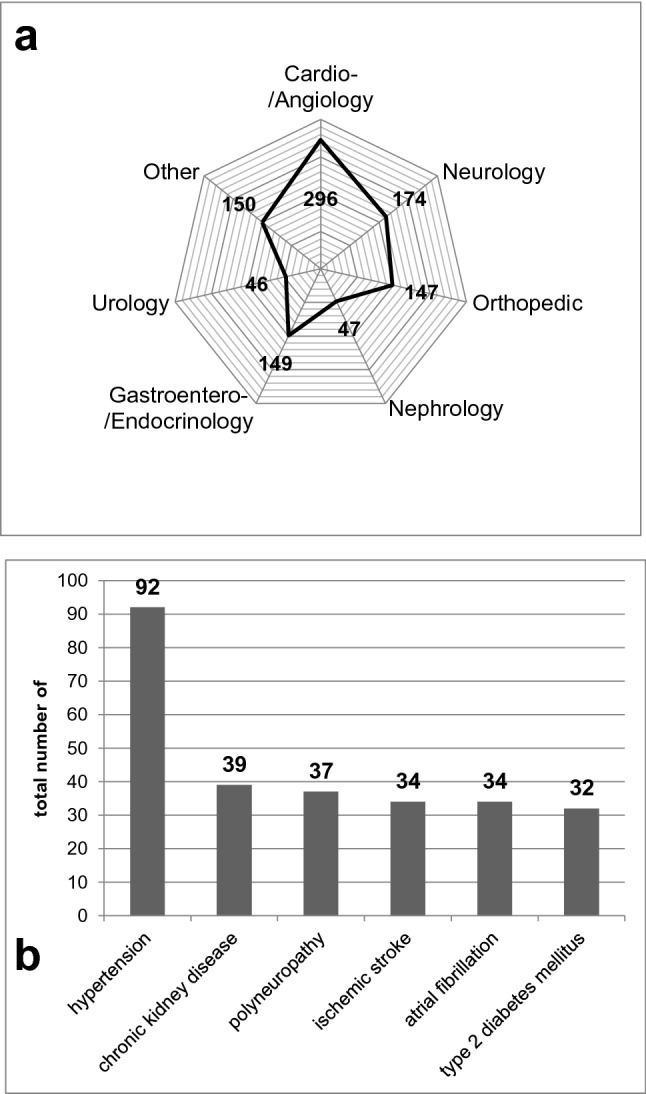
Comorbidities of geriatric PD patients (*n* = 123). **a** Shows the number of major comorbidities in different medical disciplines according to the first level of ICD-10, plotted as absolute numbers. Note, the number of comorbidities can exceed the number of patients due to co-occurrence of several comorbidities in an individual patient. **b** Shows the number of the most common comorbidities on the second level of ICD-10 as absolute numbers

Comorbidities were assigned to the respective medical disciplines according to the first level of ICD-10 (Fig. 1a). On average, the patients suffered from 8.2 ± 4.0 comorbidities. With a total number of 296 (29.3%), cardiovascular comorbidities were the most frequent, followed by neurological (*n* = 174, 17.2%), gastroentero-/endocrinological (*n* = 149, 14.8%), and orthopedic (*n* = 147, 14.6%) comorbidities. The least frequent comorbidities appeared in the disciplines urology (*n* = 46, 4.6%) and nephrology (*n* = 47, 4.7%). Comorbidities in other medical disciplines (e.g. pulmonology, psychiatry, gynecology, hematology, immunology) were summarized to “other” (*n* = 150; 14.9%). PD-related psychiatric comorbidities were collected separately (see “Patient characteristics”).

The most common comorbidities were also listed according to the second level of ICD-10 (Fig. 1b). Hypertension was by far the most common comorbidity (*n* = 92; 74.8% of all patients), followed by chronic kidney disease (*n* = 39; 31.7%) and polyneuropathy (*n* = 37; 30.1%). Both, ischemic stroke and atrial fibrillation were found in the histories of 34 (27.6%) patients; type 2 diabetes mellitus in 32 (26.0%) patients.

### FORTA prescribing pattern

**Fig. 2:**
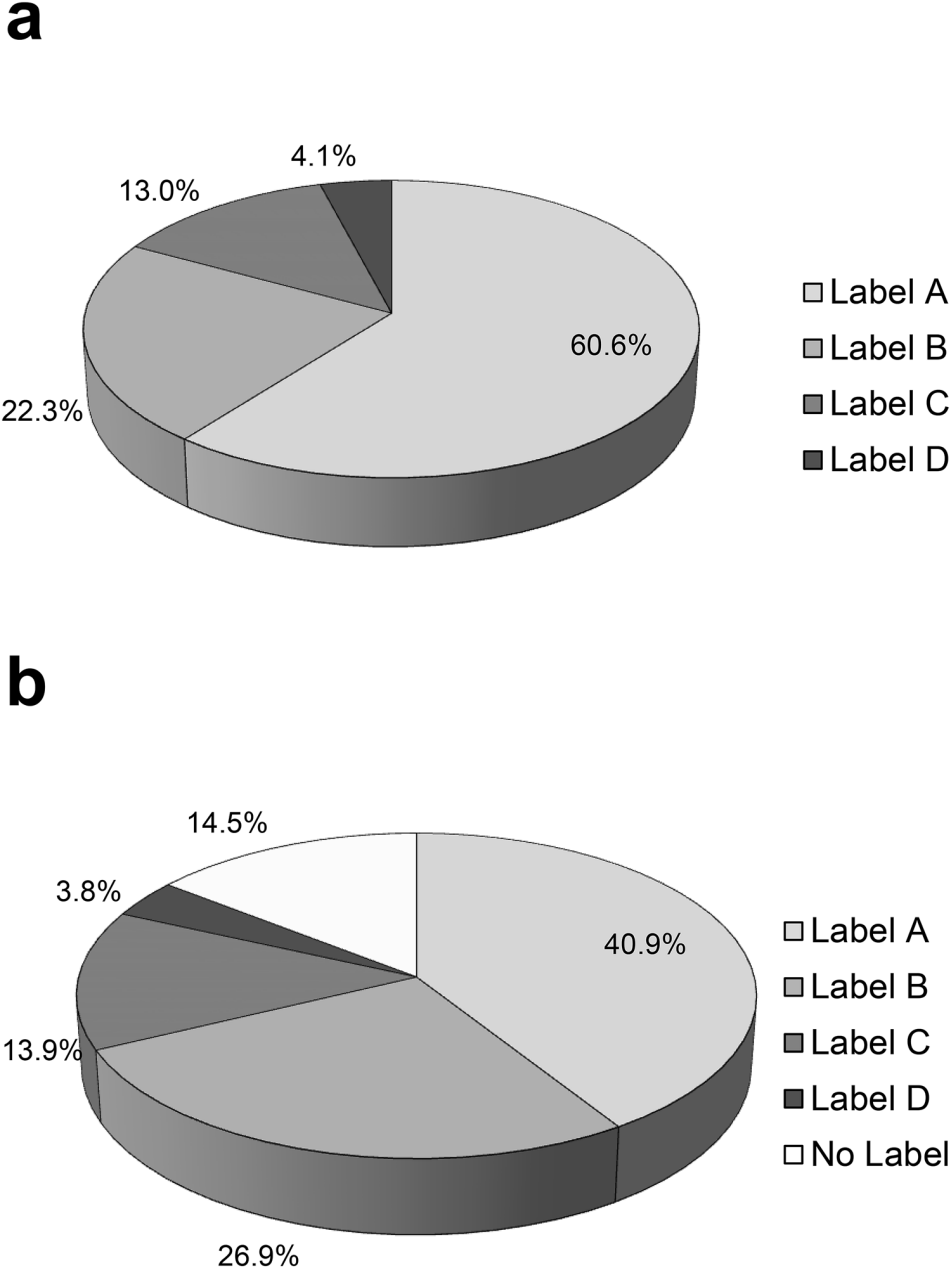
FORTA labelling of prescribed drugs.** a** FORTA labelling of PD-specific drugs in percentages of the different FORTA labels of all PD-specific drugs. **b** FORTA labelling of other drugs in percentages of the different FORTA labels of all other drugs

The analyzed patients showed considerable polypharmacy. 8.6 ± 3.4 drugs were administered per patient, subdivided into 1.6 ± 0.9 PD-specific drugs, and 7.0 ± 2.7 other drugs per patient. Figure 2a provides the proportions of PD specific drugs according to FORTA labels. 60.6% of PD-specific drugs were labeled with A, 22.3% with B. 17.1% of the drugs were questionable or should be avoided (13% label C and 4.1% label D). The most commonly administered PD-specific drug was levodopa (60.6%), followed by dopamine agonists (17.1%) and catechol-O-methyltransferase (COMT)-inhibitors (12.9%). Monoaminooxidase (MAO)-B-inhibitors (5.2%) and the NMDA-receptor-antagonist amantadine (4.2%) were used less frequently (Table 2). Amantadine was the only PD-specific drug labeled with D and was administered in eight patients (4.2%). 11 (8.9%) patients received non-oral treatment. Levodopa-carbidopa intestinal gel (LCID) was administered in 8 (6.5%) patients; 3 (2.4%) had deep brain stimulation (Table 2).

**Table 2:** PD-specific treatment

Drug	*n* (%)	Daily dose (mean ± SD) in mg	Levodopa equivalent dose (LED) according to Tomlinson et al. 2010 (mean ± SD) in mg	FORTA label
Levodopa	117 (95.1)	592.7 ± 298.8		A
Dopamine agonists				
Ropinirole	8 (6.5)	7.2 ± 5.3	144.4 ± 106.9	B
Rotigotine	10 (8.1)	8.7 ± 3.8	260 ± 114.1	B
Piribedil	3 (2.4)	183.3 ± 47.1	183.3 ± 47.1	C
Pramipexole	12 (9.8)	2.2 ± 1.3	221.7 ± 128.7	C
COMT-inhibitor				
Opicapone	1 (0.8)	50	n.a	B
Entacapone	23 (18.7)	1000 ± 363.6	330 ± 120	B
Tolcapone	1 (0.8)	300	150	n.a
MAO-inhibitor				
Rasagiline	5 (4.1)	1 ± 0	100 ± 0	C
Safinamide	5 (4.1)	70 ± 24.5	n.a	C
NMDA-antagonists				
Amantadine	8 (6.5)	212.5 ± 92.7	212.5 ± 92.7	D
Non-oral PD-specific treatment				
Levodopa-carbidopa intestinal gel (LCID)	8 (6.5)	1281.7 ± 156.1 (Data of 7 patients available)	1153.5 ± 140.5	n.a
Deep brain stimulation	3 (2.4)			
Continuous subcutaneous apomorphine (APO)	0			

40.9% of non-PD-specific drugs were labeled with A and 26.9% with B. 17.7% of the drugs were questionable or should be avoided (13.9% label C, 3.8% label D; see Fig. 2b). The most common D labeled drugs were clozapine (*n* = 11), oxazepam (*n* = 6), amitriptyline, clonidine, and lorazepam (each *n* = 2). 14.5% of the drugs administered at discharge were not included in the FORTA list for the respective indication (“no label”). The most common drugs without the recommendation in FORTA list were tamsulosin (*n* = 14), vitamin B6, allopurinol (each *n* = 8), dalteparin (*n* = 6), potassium, magnesium, midodrine, and prednisolone (each *n* = 5). Since the prevalence of lower urinary tract symptoms in the context of benign prostatic hyperplasia (as an indication for tamsulosin), hyperuricemia (as an indication for allopurinol) as well as some rheumatic/autoimmune diseases (as an indication for prednisolone) increase with age, the FORTA list still has potential for amendments and modifications.

### Frequent drug-drug interactions (DDIs)

**Fig. 3:**
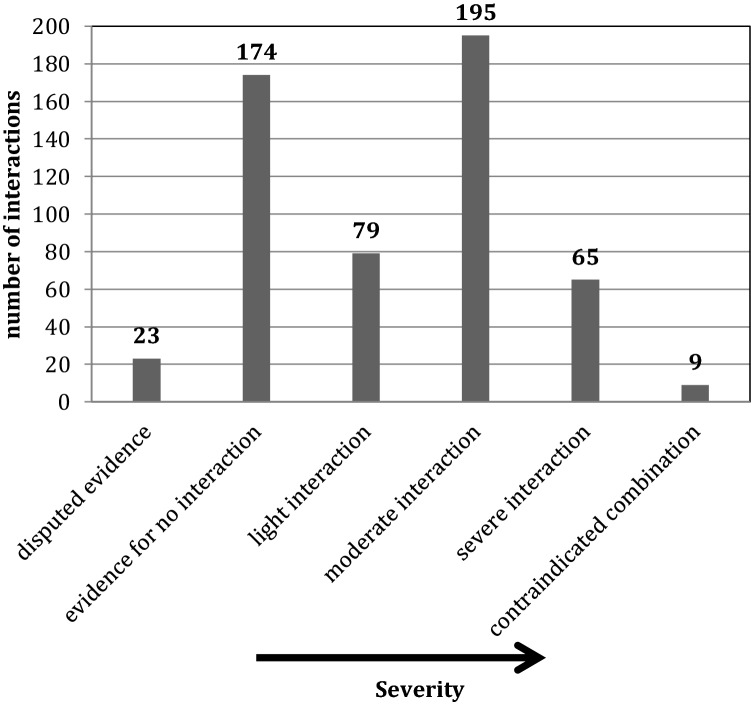
Drug-drug interactions. The figure shows the absolute numbers of interactions divided into the different categories of severity. The* x* axis lists the interactions with increasing severity from “disputed evidence” to “contraindicated combination”

Finally, the number and severity of potential DDI were analyzed (Fig. 3). In total, 545 interactions were registered, corresponding to an average of 4.4 ± 3.0 interactions per patient. The evidence for 23 (4.2%) interactions was disputed referring to the information of “Medibox”. For 175 combinations (32%), there was evidence for no unwanted interaction. 80 interactions (14.6%) were light. In contrast, 195 (35.7%) moderate, 67 (12.3%) severe interactions and 7 (1.3%) contraindicated combinations were assessed. 100 of the 123 analyzed patients (81.3%) showed at least one moderate or severe interaction, 41 patients (33.3%) had at least one severe interaction. Clozapine was involved in five of the seven contraindicated combinations (clozapine-ramipril (3), clozapine-metamizole, and clozapine-spironolactone) and in two severe interactions (clozapine-mirtazapine *n* = 2; risk for agranulocytosis). The other two contraindicated combinations were safinamide-rasagiline and tramadol-rasagiline. The most common severe interactions were between acetylsalicylic acid (ASA)-angiotensin-converting-enzyme (ACE) inhibitors/angiotensin II receptor blockers (ARBs)-diuretics (*n* = 29; reduced antihypertensive effect, deterioration in kidney function), potassium chloride-ACE inhibitors/ARBs (*n* = 7; risk for hyperkalemia), ASS-metamizole (*n* = 5; reduction of antiplatelet effect), ASS-antidepressants (*n* = 5; risk of gastrointestinal bleeding) and ACE inhibitors/ARBs-potassium-sparing diuretics (*n* = 3; risk for hyperkalemia).

## Discussion

Our cohort showed a pattern of comorbidities comparable to other geriatric PD patients and non-PD patient cohorts (Calderón-Larrañaga et al. 2016; Guisado-Clavero et al. 2018; Tönges et al. 2019). However, the pattern of comorbidities was slightly different from the study of Müller-Rebstein et al. that analyzed 127 PD patients (Müller-Rebstein et al. 2017). Surgical comorbidities were more frequent in our cohort (147 in our cohort to 64 in Müller-Rebstein et al.); psychiatric comorbidities were less common (14 in our cohort to 148 in Müller-Rebstein et al.). Moreover, the average number of comorbidities per patient in our cohort was higher than in other geriatric or PD patient cohorts (Hou et al. 2012; Müller-Rebstein et al. 2017). This could be explained by the higher complexity of patients at a university clinic and our narrow inclusion criteria (Santos-Eggimann et al. 2009; Peball et al. 2019). The numerous comorbidities highlight the importance of adequate medication in order to avoid adverse effects and DDI and, thus to reduce the risk for hospitalization (Oscanoa et al. 2017; Okunoye et al. 2020).

The FORTA classification currently includes recommendations for 296 drugs and 30 different indications (Pazan et al. 2019). With the exception of tolcapone and apomorphine, all drugs commonly used in the therapy of PD are listed. The only PD-specific drug with a FORTA label A is levodopa. Since levodopa has a favorable safety profile and the best-known effect on motor symptoms, this labeling seems reasonable referring to recent guidelines (Fox et al. 2018). Hence, levodopa is recommended as the initial treatment of geriatric PD patients (Nagayama et al. 2011; Lange and Erbguth 2017; Klietz et al. 2019).

Although dopamine agonists are classified as “*clinically useful*” in Movement Disorders Society (MDS) therapy guidelines, they are second-line drugs in elderly PD patients with multimorbidity (Mizuno et al. 2013; Hauser et al. 2014a; Fox et al. 2018; Klietz et al. 2019). The therapy studies leading to MDS recommendations, including those on patients in an advanced disease stage, were not carried out on geriatric patients. Therefore, the recommendations cannot be completely applied to a geriatric patient cohort. For dopamine agonists, the FORTA list differentiates between ropinirole and rotigotine, which are labeled with B, pramipexole and piribedil labeled C, and bromocriptine and cabergoline labeled with D. Because of serious adverse events like heart valve fibrosis, the latter is no longer used for the treatment of PD (Hubble 2002). The different labelling of the other dopamine agonists is debatable. Rotigotine, in particular, appears to be favorable in the treatment of geriatric PD patients due to its transdermal application with the continuous release. In addition, no adjustment is required in PD patients with impaired kidney or liver function (Cawello et al. 2012; Klietz et al. 2019). The dopamine agonist apomorphine is not included in the FORTA list. In our cohort, apomorphine was not administered but provides the advantage of non-oral and continuous subcutaneous application (Hagell and Odin 2001; Stacy and Silver 2008). Therefore, it can be used for the treatment of acute off-phases or motor fluctuations (Stibe et al. 1988; Dewey et al. 2001). Due to its cardiac side effects, apomorphine should be used with caution in geriatric PD patients.

COMT-inhibitors are useful for the treatment of end-of-dose-fluctuations (Tolosa et al. 2014; Ferreira et al. 2016; Fox et al. 2018). Entacapone and opicapone are labeled B in the FORTA list. The lack of a recommendation for tolcapone is probably due to its rare and second-line use as a “*possible useful*” drug in MDS therapy guidelines (Fox et al. 2018). As a result of its hepatotoxicity, the application of tolcapone requires regular controls (blood tests for liver enzymes, non-response to entacapone) (Truong 2009). In our view, the FORTA recommendations for entacapone and opicapone may be debatable. The use of opicapone may be safer for geriatric patients due to its lower potential for DDIs and less limitations for patients with liver dysfunction (Klietz et al. 2019).

The recommendations of the FORTA list for MAO-B-inhibitors do not comply with current therapy guidelines and distinguish rasagiline and safinamide, which are labeled with C, from selegiline, which is labeled with D. The selective MAO-B-inhibitors are well tolerated and showed few adverse effects in therapy studies on non-geriatric PD patients (Fox et al. 2018). Thus, Selegiline and rasagiline are recommended as initial monotherapy, rasagiline even as an adjunct therapy to levodopa (Hauser et al. 2014b; Fox et al. 2018). In contrast, the use of safinamide has only been proven as an add-on therapy for motor fluctuations (Borgohain et al. 2014). All MAO-B-inhibitors offer a considerable risk for interactions (e.g. with antidepressants). However, some studies have demonstrated a beneficial use of MAO-inhibitors and antidepressants without harmful interactions (Panisset et al. 2014; Smith et al. 2015). Although the labeling of MAO-B-inhibitors in the FORTA list appears to be somewhat drastic, they should be used with circumspection in geriatric PD patients because of the potential interactions, their limitation in the liver (all MAO-B-inhibitors) and renal dysfunction (selegiline), and the risk of other adverse effects (e.g. ulcers for selegiline) (Glavin et al. 1986; Anttila et al. 2005; Klietz et al. 2019).

The NMDA-receptor-antagonist amantadine is labeled D in the FORTA list and should be used with caution in geriatric PD patients. In addition to a large number of adverse effects, including anticholinergic adverse effects, amantadine has a high risk for DDIs, especially with other QT-prolonging drugs. Moreover, an adjustment of dose is required for patients with impaired renal function (Klietz et al. 2019). However, amantadine is approved and established as monotherapy for mildly affected de novo patients and for the treatment of levodopa-induced dyskinesia (Sawada et al. 2010; Oertel et al. 2017; Fox et al. 2018). Therefore, the use of amantadine by a specialist can be very beneficial for the patient.

Despite polypharmacy, the prescription pattern in our cohort can be reviewed as suitable for geriatric PD patients according to the FORTA classification. Still, 16.9% of the PD-specific drugs and 17.6% of the other drugs were labeled with C or D. The only PD-specific drug with FORTA label D was amantadine (eight patients, 4.1%). 28 patients took at least one non-PD drug labeled D—the most common were clozapine (in 11 patients), oxazepam (in six patients), amitriptyline, clonidine, and lorazepam (each in two patients). If possible, drugs with label D should not be used in geriatric patients due to their adverse effects, relative and absolute contraindications, and their tolerability. Avoiding these drugs is mostly possible using alternative drugs. If this is not feasible e.g. for amantadine and clozapine, a continuous risk–benefit evaluation will have to be performed for these drugs. The next sections are intended to show how PIMs for geriatric patients can be prevented using the FORTA list.

Clozapine is an atypical, second-generation antipsychotic drug and approved for the treatment of PD psychosis (PDP) (Seppi et al. 2019). In addition to non-pharmacological approaches, atypical antipsychotics, e.g. clozapine, quetiapine or pimavanserin, can be administered for the treatment of PDP (Factor et al. 2001; Cummings et al. 2014; Livingston et al. 2014; de Oliveira et al. 2015; Chen et al. 2019). Though the use of quetiapine for this indication is off-label, it seems to be a favorable alternative to clozapine for geriatric PD patients due to its similar efficacy, greater tolerability, and less serious side effects (Seppi et al. 2019; Chen et al. 2019). Pimavanserin is not approved in Germany because studies did not show an effect beyond six weeks of therapy (Meltzer et al. 2010; Cummings et al. 2014).

Benzodiazepines, e.g. oxazepam and lorazepam, and non-benzodiazepines for the treatment of sleep disorders should be avoided in geriatric PD patients, as suggested by the label D in the FORTA List (Markota et al. 2016). These drugs can result in dependency, falls, and progressive cognitive impairment (Barker et al. 2004; Hill and Wee 2012; Xing et al. 2014; Zhong et al. 2015). Accordingly, these drugs increase the risk for hospital admission and mortality (Palmaro et al. 2015; Chen et al. 2018). Extended-release melatonin, trazodone, and mirtazapine are alternative drugs for the treatment of sleep disorders in geriatric patients (Luthringer et al. 2009; Lyseng-Williamson 2012; Savarese et al. 2015). Melatonin appears to be best suited for the use in geriatric patients due to its few contraindications and excellent tolerability, therefore it is recommended in the MDS therapy guidelines (Seppi et al. 2019).

Tricyclic antidepressants, e.g. amitriptyline, are recommended for the initial treatment of depression in PD (Deuschl et al. 2020; Seppi et al. 2019). The selective serotonin reuptake inhibitors (SSRIs), e.g. sertraline, citalopram, escitalopram, show an effect equivalent to amitriptyline but have less severe interactions and fewer adverse effects (Thorlund et al. 2015; Beyer and Johnson 2018). According to the FORTA list, SSRIs should be considered in geriatric patients, especially sertraline with regard to the favorable clinical study results (Bose et al. 2008; Seitz et al. 2010; Thorlund et al. 2015).

Finally, it should be highlighted that the FORTA list contains general prescription recommendations for geriatric patients based on expert opinions. The results of our study could motivate not specialized general practitioners and resident neurologists to use the FORTA list to increase drug-safety in geriatric PD patients. In addition, it should be emphasized that geriatric PD patients are primary cared for by these practitioners after their discharge. A considerable proportion of patients change their medication after discharge from a hospital. A third of these patients change their medication on their own due to side effects, missing effect of the medication, missing knowledge about the indication, running out of medication, or nonspecific reason (Feldmann et al. 2020). The FORTA list can possibly be helpful for choosing a favorable medication and prevent from such medication changes. Still, the administration of specific drugs for certain indications by PD specialists, who have extensive experience with the drugs, can still be beneficial for the patients. We do not think that this list would be an appropriate tool for these specialists.

The number of administered drugs correlates directly with the number of DDIs (Dias et al. 2019). The proportion of patients with at least one moderate (72.6% versus 26% in Johnell et al. or 27.5% in Sánchez-Arenas et al.) or severe interaction (34.7% versus 5% in Johnell et al. or 7.9% in Sánchez-Arenas et al.) in our PD cohort was higher than in other cohorts (Johnell and Klarin 2007; Sánchez-Arenas et al. 2012). On the one hand, this was probably due to our narrow inclusion criteria selecting a cohort of patients with extensive polypharmacy. These studies analyzed elderly patients and did not exclusively include PD inpatients with two or more comorbidities and at least five administered drugs at discharge. On the other hand, the greater number of administered drugs in our cohort could be the reason for the higher amount of relevant interactions. The complex treatment of PD and associated comorbidities often requires the administration of several different drugs, thus leads to considerable polypharmacy and provides higher interaction potential (McLean et al. 2017; Müller-Rebstein et al. 2017; Csoti et al. 2019).

Five of seven contraindicated combinations were associated with the administration of clozapine (in combination with metamizole, spironolactone or ramipril) and resulted in a significantly higher risk of life-threatening agranulocytosis (de la Chapelle et al. 1977; Krupp and Barnes 1989). The other two contraindicated combinations were rasagiline-safinamide and rasagiline-tramadol. In general, the combination of different MAO-inhibitors (e.g. rasagiline and safinamide) is contraindicated due to the significant risk for adverse events, namely the hypertensive crisis and serotonin syndrome. DDIs can result in serious adverse drug effects, hospital admissions, and higher mortality (Hines and Murphy 2011; Pasina et al. 2013; Dechanont et al. 2014). Therefore, to avoid possible harmful DDIs, the medication should be tested for interactions at the start of a new drug and at regular intervals during treatment using commercially available software or databases (Moura et al. 2012; Roblek et al. 2015). If clinically relevant DDIs are unavoidable to achieve an adequate therapeutic effect, sufficient laboratory and/or clinical monitoring should be performed.

## Limitations

We present mono-centric data from a large inpatient cohort of geriatric PD patients characterized by multimorbidity and polypharmacy. These data were collected retrospectively. A minor number of drug indications were not clarified from the original documentation and had to be analyzed by the meta-data of the hospital stay. In these cases, the drug was assigned to a FORTA label (see “Analysis”). We also cannot give any information on the actual patient safety outcomes due to the retrospective nature of the study design. This issue has to be addressed in future clinical trials. The FORTA list is updated at regular intervals and drugs are added each time. Accordingly, the recent FORTA list does not yet include all possible drugs for all indications, such as drugs for the treatment of orthostatic hypotension (midodrine) or symptoms of the lower urinary tract due to prostate hyperplasia (tamsulosine). We will contact the FORTA expert group and suggest including these drugs in the next recommendations. Our literature research revealed that there is a lack of real-life data on actual complications as a result of the administration of contraindicated or interacting drugs for geriatric PD patients. The plans of our study group are to meet the existing demand for longitudinal, prospective studies on this topic.

## Conclusions

This is the first study investigating the appropriateness of the FORTA list for a large inpatient cohort of geriatric PD patients. The recommendations of the FORTA list for PD-specific drugs are reasonable for geriatric PD patients, though tolcapone and apomorphine were not mentioned in FORTA list. When applying the list on geriatric inpatients, the observed prescribing pattern was predominantly safe and adequate. Nevertheless, there were still a noticeable number of PIM and DDI that may affect patients’ safety and might be avoided using alternative drugs. This study highlights the importance of high awareness, sufficient education, and preventive interventions for treating physicians of geriatric PD patients to increase drug safety. The FORTA concept displays a potential tool for preventive interventions. However, since the FORTA list publishes rather general recommendations for general practitioners, poorly labeled drugs can still be of great benefit for geriatric patients when prescribed by specialists taking appropriate precautions.
